# Women and Substance Abuse Problems

**DOI:** 10.1186/1472-6874-4-S1-S8

**Published:** 2004-08-25

**Authors:** Renée A Cormier, Colleen Anne Dell, Nancy Poole

**Affiliations:** 1 British Columbia Centre of Excellence for Women's Health, E311-4500 Oak Street, Vancouver, Canada; 2 Canadian Centre on Substance Abuse, 75 Albert Street, Ottawa, Canada; 3 British Columbia Centre of Excellence for Women's Health, E311-4500 Oak Street, Vancouver, Canada

## Abstract

**Health Issues:**

Differences exist in the prevalence and physical health impacts of problem substance use among men and women. These differences are also found in the mental health and trauma events related to substance use, barriers to treatment and harm-reduction services and the impact of substance use on pregnancy and parenting. Data from the 2000–2001 Canadian Community Health Survey and Canada's Alcohol and Other Drugs Survey (1994) were used to explore this issue further.

**Key Findings:**

While women use alcohol and illicit drugs at lower rates than men, the health impact of their use is significant, and in some cases greater than for men. Women are more likely to use prescribed psychoactive drugs (e.g. pain relievers, sleeping pills, tranquillizers) and most of these drugs have addictive potential and long-term negative consequences on health. Research collected from treatment centres in Canada show high rates of victimization experienced by women, which have implications for both their substance use treatment and improvement in mental health.

**Data Gaps and Recommendations:**

Significant gaps exist in our knowledge on the level, type, and impact of substance use and the adequacy of programming for Canadian women. Information that might be used to guide prevention initiatives, such as the amount of alcohol that might safely be used in pregnancy and the incidence of fetal alcohol syndrome, related birth defects and developmental disabilities are unknown. Improved surveillance, sensitive and comprehensive screening for substance use problems, accessible treatment and harm reduction programming, and coordination with the mental health and violence fields are recommended.

## Background

It is particularly challenging to provide policy-relevant information on women in the area of substance use and misuse, as the topic covers such broad territory. Problem substance use has been found to arise from a complex interplay of biological, genetic, psychological, social, cultural, relational, environmental and spiritual factors. Because of the interplay of these factors, it remains a challenge for policy makers and program planners to develop and implement the very broad, collaborative, systemic responses required and to do so in a manner that links prevention, enforcement, harm reduction and treatment strategies. In spite of the overall challenges, advances in gender-specific policy and programming are especially worthy of consideration and could be of tremendous benefit to the health of women and their families.

This chapter presents findings from both surveillance sources and select research reports. As there has not been recent or adequate surveillance specific to substance use by Canadians, cross-sectional data from the Canadian Community Health Survey (CCHS) Cycle 1.1 (2000) are highlighted. While trends in women's substance use and substance use problems could not be provided, an overview of key research findings is offered that supports a comprehensive understanding of the topic from a gender perspective and places the findings of the CCHS in the appropriate context. Readers are encouraged to consult existing research reports mentioned throughout the chapter to further their understanding of this complex topic.

### Physical Health Impact of Problem Substance Use

Health Canada's Straight Facts about Drugs and Drug Abuse [1] outlines the health consequences of high doses of all classes of psychoactive substances, including stimulants, hallucinogens, cannabis and central nervous system depressants (opioid analgesics, alcohol, inhalants/solvents, benzodiazepines, barbiturates).

For some drug classifications, evidence of sex differences in the physical health impact of substance use is available. [2] To take alcohol as an example, women metabolize alcohol and other psychoactive substances more slowly than men, allowing harmful metabolites to remain longer in the body. Women are more likely than men to develop cirrhosis of the liver after consuming lower levels of alcohol over a shorter period of time. [3,4] These findings also apply to brain shrinkage and impairment, [5-7] breast cancer, [8-12] gastric ulcers [13] and alcoholic hepatitis. [14]

Although there is not adequate research on sex differences in the impact of illicit drugs, gender differences are beginning to be documented. [15] Injecting drug use (IDU) is a key risk factor among women for the transmission of blood-borne diseases such as HIV/AIDS and hepatitis C. [16] Also linked to illicit drug use are high-risk sexual behaviours (e.g. sex trade work), which, in turn, are associated with a range of negative health impacts. [17]

### Mental Health, Trauma and Substance Use

Research has shown that as many as two thirds of women with substance misuse problems may have a concurrent mental health problem, such as depression, post-traumatic stress disorder, panic disorder and/or an eating disorder. [18] Research also shows that a large proportion of women with substance use problems are victims of domestic violence, incest, rape, sexual assault and child physical abuse. [19-22] Victimization has been linked to a variety of negative outcomes, including post-traumatic stress disorder, depression, anxiety, suicidal behaviour and low self-esteem among women in the general population. [23,24] Compared with non-abused clients, women in treatment for problem substance use who have been victimized are more likely to suffer from depression and suicidal ideation, have lower self-esteem, negative psychological adjustment and more post-traumatic stress symptoms. [25-30]

### Pregnancy, Mothering and Substance Use

Substance use by pregnant women and mothers has received a great deal of attention, and there is a voluminous literature documenting the adverse effects of alcohol, tobacco and other drugs on the fetus. Alcohol use during pregnancy, particularly in combination with poor nutrition, poor general health, experience of trauma and mental health problems, and lack of prenatal care, has been found to have the most harmful effects. [31,32]

Maternal use of licit and illicit drugs can also result in problems that have short- or long-term consequences for those prenatally exposed. However, study of the impact of these drug categories is hampered by methodological flaws that fail to take into account the use of more than one drug. [33] Rather than the provision of effective outreach, engagement and treatment, the responses in some countries and to some extent in Canada towards women who use illicit substances during pregnancy have been blame, attempts to force women into treatment, and even criminal sanctions. [34-37]

The Health Canada document entitled Best Practices: Fetal Alcohol Syndrome/Fetal Alcohol Effects and the Effects of Other Substance Use during Pregnancy [32] provides a comprehensive view of the issues and of promising fetal alcohol syndrome (FAS) prevention practices grounded in both the literature and the expertise of Canadians working in the field. Programs such as the Breaking the Cycle Program in Toronto and the Sheway Program in Vancouver are examples of effective programs being developed in Canada to serve women at high risk of having a child affected by FAS.

### Stigma and Barriers to Treatment for Women

In the forefront of psychosocial influences on women's misuse of alcohol and other drugs is the stigma arising from societal attitudes towards women's substance use. [38] This stigma causes women to feel considerable guilt and shame as their substance use/misuse continues and creates barriers to their accessing help. [39-43] The stigma associated with women's substance use affects service providers as well. Women often encounter mis-information, denial, inaction and even punitive attitudes towards their substance use by professionals in a position to intervene, and thus they may not be identified as needing help. [37,44-46]

Stigma intersects with structural and other barriers that arise from experience of victimization and mental health problems, fear of having one's children apprehended, minority status, income and rural location, to name but a few. Health Canada's report Best Practices: Treatment and Rehabilitation of Women [40] has been developed as a key resource for health care providers in helping to reduce barriers to treatment access for diverse groups of substance-using women.

The barriers to accessing supportive treatment services are even greater for pregnant and parenting women. Programs that accept both mothers and children are very scarce. Finding affordable and safe care for their children as well as residential care is often an overwhelming obstacle for mothers needing treatment. [47] Although outpatient counselling and other supports for pregnant and parenting women have been made available, appalling barriers remain for mothers in need of residential treatment for problem substance use in Canada.

### Gender and Harm Reduction Approaches

Harm reduction is a newer policy approach to substance misuse, which arose initially as a response to the spread of AIDS among injection drug users. [48] The primary goal of harm reduction policy and practice is a reduction in the negative consequences associated with use rather than complete cessation of substance use. Key harm reduction programs and policies in Canada relating to illicit drugs include methadone maintenance; needle/syringe exchange; supervised injection sites; increased attention to the decriminalization of use of small quantities of cannabis; and education, information and diversion programs. Such initiatives can be beneficial to women who are drug users and/or who have partners who are drug users in helping prevent HIV transmission, improve their access to drug treatment and health care services, prevent birth defects and disabilities in their children, make incarceration experiences safer, and stabilize their health overall. Particularly promising is harm reduction programming offered to high-risk, marginalized pregnant women and their support networks, an approach that has been shown to prevent FAS and other alcohol- and drug-related disabilities, support retention of custody, and increase the health and social stability of both mothers and children. [47]

### The Current Study

We explored the use of alcohol, licit and illicit substances by Canadian women and subgroups of women (including girls, elderly women, Aboriginal women and pregnant women). Additionally, the health consequences of substance use, and the coexistence of mental health, trauma and substance use issues, were explored.

## Methods

### Data

Cross-sectional data from the Canadian Community Health Survey (CCHS) Cycle 1.1 (2000–2001) [49] are presented below. The data are weighted to represent the Canadian population. Age-adjusted data are presented where appropriate. The sample comprised 125,574 individuals, aged 12 years and older, from across all provinces and territories. Data from other sources (including select research reports, such as the Canadian Profile on Alcohol, Tobacco and Other Drugs [50]) are similarly provided to highlight what is known about the use of alcohol, licit and illicit drugs by women, and the effects of this use. Where pertinent, comparisons between females and males are made.

It is important to note that comparability issues arise when different surveys, data sources and research reports are compared. Key issues of concern are related to design (e.g. the jurisdictions included in the survey) and methodology (e.g. the form of the survey – telephone or face-to-face interviews). For a full understanding of the comparability of the research studies reported on here, the reader is encouraged to consult the original source. Likewise, it is important to point out some of the key limitations of general population surveys. These include the difficulty in obtaining information from and about hard-to-reach populations (heavy substance users living on the street or in an institution), under-reporting because the behaviour is socially sanctioned, recall difficulties, and/or under-reporting of psychoactive medication use among older adults due to their lack of awareness of the reasons for taking multiple medications. Nonetheless, survey instruments are able to provide information regarding the lower-bound estimates of substance use measures important to public health monitoring. [50]

### Measures

#### Alcohol Use

Data from the Alcohol (and Breast-feeding) modules of the CCHS were extracted in order to determine lifetime use of alcohol, alcohol use and frequency of use in the previous 12 months, heavy drinking (defined here as five or more drinks on at least one occasion at least once per month), and alcohol use and frequency of use in last pregnancy. Differences by sex, age and Aboriginal status were explored. Data examined by Single and colleagues from 1994 to 1995 and 1995 to 1996 from the Canadian Institute for Health Information (CIHI) on hospital separations attributed to alcohol use are strongly endorsed within the field and are also presented. [51]

#### Licit Drug Use

Data from the Drug Use module of the CCHS were extracted in order to determine the use of the following licit substances: pain relievers, tranquilizers, diet pills, antidepressants, opioid analgesics (such as codeine, Demerol or morphine), and sleeping pills. Only participants from Ontario (all health regions) were administered the Drug Use module of the CCHS. As a result, findings may not be generalizable to other provinces and territories. Differences by sex, age and Aboriginal status were explored. The literature on the health consequences of long-term licit drug use is summarized.

#### Illicit Drug Use

Information about illicit drug use was not collected through the CCHS Cycle 1.1, therefore data from the 1994 Canada's Alcohol and Other Drugs Survey (CAODS),[52] the 1998 Canadian Campus Survey [53] and hospital separations attributed to illicit drug use [51] are presented.

#### Mental Health Issues, Trauma and Substance Use

Data from the Depression, Contacts with Mental Health Professionals, and Suicidal Thoughts and Attempts modules of the CCHS Cycle 1.1 were extracted in order to explore the relation between mental health issues and substance use in women. Seventy of the 136 health regions in Canada (all in the provinces of Nova Scotia, New Brunswick, Quebec, Manitoba, Alberta, and a few in Ontario) administered the Suicidal Thoughts and Attempts questionnaire. All but two health regions administered the Depression questionnaire. Data concerning rates of trauma among substance-using women in treatment centres in Canada are presented.

## Results

### Alcohol Use

#### Prevalence

**Figure 1:**
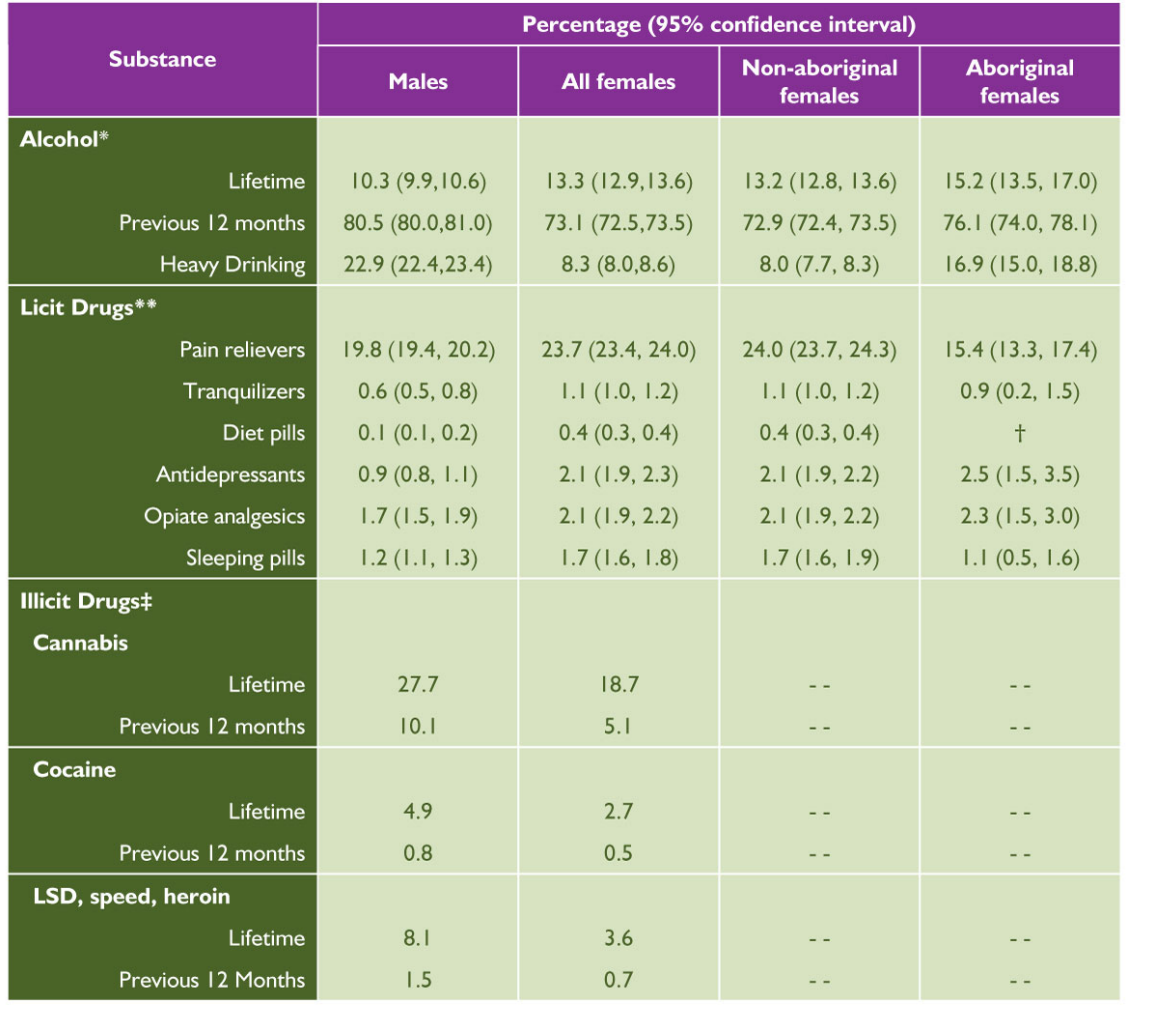
Prevalence of Alcohol Use, Licit Drug Use and Illicit Drug Use in Canada by Sex and Aboriginal Status

**Figure 2:**
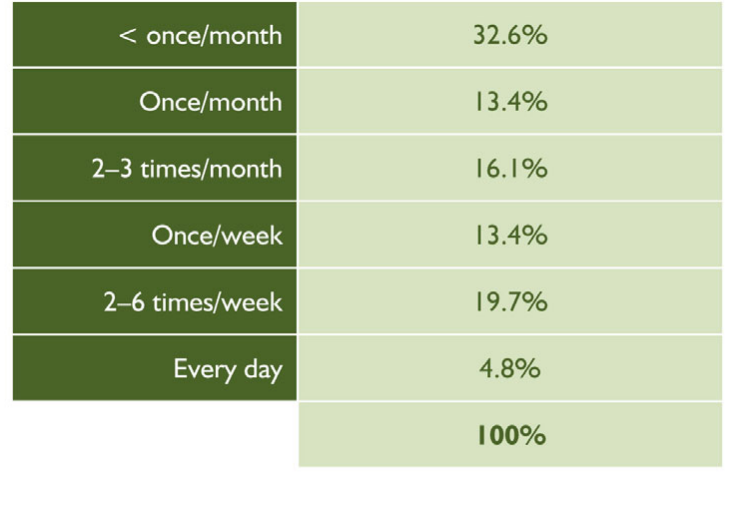
Patterns of Alcohol Consumption among Women who Reported Having had a Drink in the Previous 12 Months: Canadian Community Health Survey [49]

According to the 2000–2001 CCHS, [49] 73.1% of Canadian women aged 12 years and older had used alcohol at least once in the previous year and 13.3% reported using alcohol during their lifetime but not in the previous year (see Figure 1). Alcohol use was more prevalent among males than females. The majority of females who reported having had a drink in the previous year tended to drink infrequently (see Figure 2).

The rate of alcohol use in the previous 12 months among adolescent girls aged 15 to 19 years was 71.1% and among Canadian women, on average, was 75.5%. It appears that more Aboriginal than non-Aboriginal women reported ever drinking alcohol and drinking alcohol in the previous 12 months. Roughly 11% of the women reporting alcohol use in the previous 12 months met the criterion for any occasion heavy drinking. Twice as many Aboriginal women as non-Aboriginal women met this criterion.

#### Alcohol Use in Pregnancy

Fourteen percent of all women who indicated that they consumed alcohol in their lifetime also reported that they had consumed alcohol during their last pregnancy. The vast majority of women reporting alcohol use during a pregnancy reported drinking infrequently: 75.4% drank less than once per month; 9.7% drank once per month; 6.5% drank two to three times per month; 5.3% drank once per week; and 1.3% drank every day.

#### Morbidity

Single and colleagues [51] examined 1994–1995 and 1995–1996 data from CIHI and 1991–1992 data from the then Laboratory Centre for Disease Control, Health Canada, on hospital separations attributable (fully and partially) to alcohol (e.g. alcohol toxicity, alcoholic liver cirrhosis) and estimated that 29,181 women (51,765 men) were hospitalized in 1995–1996 because of alcohol. The greatest number of hospitalizations among women was for accidental falls (7,689), followed by motor vehicle accidents (3,433), alcohol dependence syndrome (3,247) and suicide/self-injury (2,473). Hospital separations per 100,000 have remained fairly stable among women (from 206 in 1991–1992 to 193 in 1995–1996) and have declined among men (from 401 in 1991–1992 to 349 in 1995–1996).

#### Mortality

According to Single and colleagues' analysis of 1995 CIHI data, the estimated number of deaths attributed (fully and partially) to alcohol consumption among hospitalized women was 1,823 or 12.4 per 100,0000 population (among men it was 4,681 or 32.3 per 100,000 population). The greatest number of deaths among women was attributed to motor vehicle accidents (357), alcoholic liver cirrhosis (257), accidental falls (202), breast cancer (192), alcohol dependence syndrome (135) and suicide/self-inflicted injury (134). The potential years of life lost in 1995 were estimated at 48,392 for women or 327.9 per 100,000 population (123,734 for men or 853.7 per 100,000 population).

### Licit Drug Use

**Note: **The licit drug use identified in the CCHS survey should not be seen as indicating misuse or problemuse.

#### Prevalence

According to the 2000–2001 CCHS, the proportion of females aged 12 or older reporting use in the previous year of selected prescription and non-prescription drugs was 23.7% for pain relievers, 2.1% for opioid analgesics, 1.7% for sleeping pills, 1.1% for tranquilizers, 2.1% for antidepressants and 0.4% for diet pills. Females consistently reported higher rates of use than males in all categories (see Figure 1). Non-Aboriginal women were more likely than Aboriginal women to report the use of pain relievers and sleeping pills.

#### Morbidity

Women were twice as likely as men to have benzodiazepines prescribed to them for "non-clinical" symptoms, [54] such as stress from work or home life, grief, acute or chronic illness, physical pain or adjustment to a major life change, [55-57] and to have them prescribed for longer periods. [58] It is becoming clear that women are over-prescribed benzodiazepines to cope with difficult life circumstances rather than to relieve severe clinical symptoms. Long-term benzodiazepine use is associated with several negative health consequences. The sedative/hypnotic effects of these drugs place elderly individuals at increased risk of psychomotor, cognitive and memory impairment; [59] emotional clouding; violent behaviour; depression; [60] and hip and femur fractures. [61,62]

### Illicit Drug Use

#### Prevalence

Overall, self-reported illicit drug use by females in Canada is low (see Figure 1). Data from the 1994 CAODS [52] show that the proportion of females aged 15 and older reporting use of selected illicit drugs in the previous year was 5.1% for cannabis, 0.7% for LSD, speed or heroin, and 0.5% for cocaine. When these proportions are compared with data on lifetime use of illicit drugs from the same survey, the rates of use in each of the three categories decrease slightly: 18.7% for cannabis, 3.6% for LSD, speed or heroin, and 2.7% for cocaine. More recent data (1998 Canadian Campus Survey [53]) reveal comparatively high rates of illicit drug use among university students (although these figures do not represent the use among Canadians in general): 8.9% of female students reported use of illicit drugs in the previous 12 months (not including cannabis) and 28.0% reported cannabis use. Illicit drug use by males was higher than by females inall categories.

**Figure 3:**
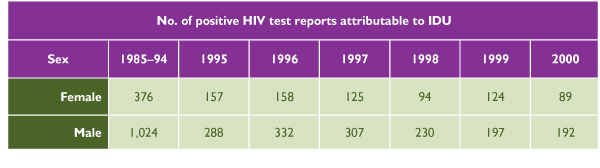
Number of Positive HIV Test Reports Attributable to Injecting Drug Use (IDU) among Adult Females and Males 15 Years of Age and Older, by Year of Test [16]

Injecting drug use (IDU) provides a good illustration of the need for a gender-based analysis of illicit drug use. Surveillance data from the Centre for Infectious Disease Prevention and Control, Health Canada, [16] reveal that up to December 31, 2000, IDU was identified as the risk factor in 1,123 reported positive HIV cases in women and in 2,570 cases in men. Figure 3 shows that, among both females and males, there has been a decrease over time in the number of positive tests attributable to IDU. However, IDU is a greater risk for females, accounting for an average of 45.7% of all female HIV cases and only 25.6% of all male cases from 1995 to 2000. Over this six-year period there was a substantial decrease of IDU as a risk factor for HIV among females (from 53.6% cases in 1995 to 38.5% in 2000), whereas among men the rate remained relatively stable (from 23.6% cases in 1995 to 22.4% in 2000).

There are extremely limited Canadian data on solvent use. Information from the CAODS shows that 0.3% of females aged 15 and older reported use of a solvent(s) throughout their lifetime. The Ontario Student Drug Use Survey [63] reported that between 1977 and 2001, the average prevalence of glue sniffing with the intention of getting high among females during the previous 12 months was 2.1%; the percentage was highest in 1999 (3.7%) and lowest in 1991 (1.1%).

#### Morbidity

From their analysis of hospital separations attributed (fully and partially) to illicit drugs, Single and colleagues estimated that 2,405 women (4,542 men) were hospitalized in 1995–1996 because of illicit drugs. [5] The hospitalizations for women were a result of drug psychoses (768), followed by cocaine dependence or abuse (375), psychotropic poisoning (323), and opioid dependence/abuse (314). The number of hospital separations per 100,000 population has remained fairly stable between 1991–1992 and 1995–1996 for women and men.

#### Mortality

According to Single and colleagues' analysis of 1995 CIHI data, the number of deaths attributable to illicit drugs was estimated at 108 for hospitalized women or 0.7 per 100,000 population (695 for men or 4.8 per 100,000 population). The greatest number of deaths resulted from opiate poisoning (42), poisoning (intent undetermined) (23), AIDS (18), suicide/self-inflicted injury (15) and cocaine poisoning (11). The potential years of life lost in 1995 were estimated at 4,959 for women or 33.6 per 100,000 population (28,710 for men or 198.1 per 100,000 population).

### Mental Health, Trauma and Substance Use

#### Mental Health and Substance Use

Of the women meeting the criterion for heavy drinking, 25.7% reported feeling sad, blue or depressed in the previous two weeks. Depression can be a precursor or antecedent of problem alcohol use, or both. Roughly 15% of heavy drinking women also reported visiting a mental health professional in the previous 12 months. Further, 18.6% of them reported that they had previously seriously considered committing suicide. Of the women who had considered suicide in the previous 12 months, 37.3% had previously attempted to take their own life.

#### Trauma and Substance Use

Research shows high rates of victimization among substance-abusing women. [15] Data collected from substance use treatment centres in Canada corroborate these estimates. In a sample of substance-abusing women from nine treatment centres across Ontario, Cormier [64] found that 85.7% of the 98 women in her sample had been victimized. They were most likely to have reported being a victim of adult physical abuse (56.1%), childhood sexual abuse (56.3%), childhood physical abuse (56.1%) and adult sexual abuse (45.4%). A treatment facility for substance-abusing women in Vancouver (Aurora Centre) reported that 65% of its female clients had been physically assaulted as adults, and 38% had been sexually assaulted; 47% reported physical violence in childhood, and 53% reported sexual abuse at that time. [47]

## Discussion

### Data Limitations

The past quarter-century has been characterized by inconsistent survey data collection on substance use and abuse at the national, provincial/territorial and municipal levels. Further, there has been limited recognition in the research that social factors specific to sex and gender (e.g. employment, cultural displacement, abuse) have a significant role in substance use problems. An overarching limitation of most surveys and research studies is the marked discrepancy in definitions of substance use, dependence, or problematic use.

In comparison with current information on alcohol use and abuse in Canada, there is extremely limited knowledge of illicit and licit drug use and, in particular, the associated morbidity and mortality. Gender-specific research on prescription and non-prescription drug use is required, since females consistently report higher rates of use than males, thus increasing the probability of problematic use. Health Canada and pharmaceutical company data (e.g. PURDUE Pharma) are alternative sources of information on the dispensing practices of physicians. In addition, there has been collection of drug plan utilization data at the provincial level (e.g. New Brunswick, Saskatchewan) that could be replicated. Further, while the first cycle of the CCHS Cycle 1.1 did not include questions about illicit drug use, the most recent cycle of the survey (CCHS Cycle 1.2) will be an appropriate source from which to collect this information.

There are also knowledge gaps concerning substance use and misuse, and concerning competent programming that addresses the needs of poor women, rural women, homeless women, women with disabilities, lesbians, elderly women, adolescent girls and women from different ethnic backgrounds, all of whom are often at increased risk for general health problems. The limited data available suggest that these subpopulations of women may be at greater risk of substance use problems than the general population. [65-73]

Several suggestions to address the current data limitations follow. Each of these requires the collection of sex- and gender-specific data.

## Recommendations

### Incidence and Prevalence Data

1. Surveillance in the form of a national incidence/prevalence survey should be carried out on a regular basis (e.g. every three years).

2. Canada should provide more support for its substance use monitoring systems, such as the Canadian Community Epidemiology Network on Drug Use, [74] so that data will be readily available and attempts to collate data in reports such as this one will not be such a daunting task. Integral to such a system must be the reporting of data by sex, attention to social and other determinants of women's substance use, and the exploration of substance use problems in relation to gender.

### Treatment Data

3. There is a need to develop a mechanism for capturing evaluation data on substance use interventions (including women-specific interventions at various levels of care).

4. There is also a need to examine the availability of data collected from special populations (e.g. National Youth Solvent Abuse Program, First Nations and Inuit Health Branch).

### Mortality and Morbidity Data

5. Greater and affordable access to national systems for collecting and reporting information on hospitalizations is necessary (i.e. CIHI); all aspects of the standardization in data collection must be addressed (e.g. ICD-9/10 codes); and hepatitis C in addition to HIV/AIDS should be included.

### Other Data Fundamental to Women-Centred Policy and Programming

6. Central to action on women's substance use is addressing the misinformation and prejudice directed at women with this common health-related problem. This is essential in order to help reduce the guilt and shame that prevent women from identifying both that they use substances and that they need help. This approach should underlie all the following recommendations.

### Reducing Barriers to Identifying and Preventing FAS

7. There is a need to identify viable and sensitive methods for implementing screening for substance use by women on the part of a wide range of professionals in a position to make referrals to treatment and other resources when necessary. It is particularly important for this screening to be in place to reach women of child-bearing years and pregnant women, so that FAS and other alcohol- and drug-related developmental disabilities are prevented. Some surveys on alcohol and drug screening practices of physicians have been done, and such surveys can assist in promoting tailored training and action in this important area.

### Understanding and Responding to Diverse Needs

8. Knowledge gaps need to be addressed concerning the level, type and impact of substance use and the adequacy of programs that should be reaching vulnerable subgroups of women (e.g. Aboriginal women, poor women, homeless women, lesbians, women living in rural areas). For the most part, these would require smaller-scale, local studies.

### Understanding the Impact of Illicit Drug Use on Women and Harm Reduction Approaches

9. There is a critical need for data on sex and gender differences in the experience of illicit drug use and the potential need for, and impact of, harm-reduction-oriented policy and programming.

### Improving Treatment Access, Especially for Mothers

10. Data on the impact of barriers to treatment are needed. Treatment programming that addresses sex and gender differences in the experience of addictions needs to be made more accessible to women in Canada. Programming that is accessible and relevant to women who are mothers is of particular priority.

### Blending Treatment/Support in Substance Use, Mental Health and Trauma Concerns

11. It is recommended that linkages be enhanced (and in some cases program integration be considered) between mental health treatment, substance misuse treatment and programming for women who have experienced trauma/relationship violence, in order to address the strong interconnections among these three serious health problems for women.

## Notes

The authors wish to acknowledge the contributions of Bette Reimer, Canadian Centre on Substance Abuse, who conducted literature searches for this chapter, and Karen Devries, who assisted with the organization of the reference section for this chapter.
